# CRISPR-Cas13a Based Visual Detection Assays for Feline Calicivirus Circulating in Southwest China

**DOI:** 10.3389/fvets.2022.913780

**Published:** 2022-07-11

**Authors:** Jian Huang, Yunjia Liu, Yuwei He, Xiaonong Yang, Yan Li

**Affiliations:** ^1^Department of Veterinary Medicine, College of Animal Husbandry and Veterinary Medicine, Southwest Minzu University, Chengdu, China; ^2^Veterinary Teaching Hospital of Southwest Minzu University, Chengdu, China

**Keywords:** Crispr-cas13a, RPA, visual approach, nucleic acid detection, feline calicivirus

## Abstract

Feline calicivirus (FCV) is a well-known causative pathogen for upper respiratory infection in cats. Its high genetic variability challenges existing molecular diagnostic methods in clinical settings. Thus, we developed two sensitive and visual assays for FCV nucleic acid detection based on RPA reaction and CRISPR-Cas13a trans-cleavage activity. Recombinant plasmid DNA, crRNAs, and RPA primers were designed and prepared, respectively, targeting to FCV ORF1 gene. Besides, purified LwCas13a protein was produced by *E.coli* prokaryotic expression system. To confirm the validity of FCV-Cas13a assays, seven reaction systems (RSs) with different components were tested, and visual readouts were displayed by lateral flow dipstick (FCV-Cas13a-LFD) and fluorescence detector (FCV-Cas13a-FLUOR), respectively. The established FCV-Cas13a assays were capable of detecting FCV nucleic acid in presetting RSs without cross-reaction with other feline-associated pathogens, and the detection limit was as low as 5.5 copies/μl for both visual methods. Moreover, the positive rate of 56 clinical specimens detected by FCV-Cas13a assays (67.9%, 38/56) was notably higher than that of RT-qPCR (44.6%, 25/56) (*p* < 0.001), including 13 presumptive positive specimens. Taken together, FCV-Cas13a assays provided reliable and visual diagnostic alternatives for FCV field detection.

## Introduction

Feline calicivirus (FCV) is one of the causative pathogens of upper respiratory tract disease in cats, mainly presenting with oculonasal discharges, and chronic gingivostomatitis (1). As described in previous studies, FCV is a single-stranded, non-enveloped RNA virus belonging to the genus *Vesivirus*, family *Caliciviridae*, and its genome contains three open reading frames (ORFs) that encode ORF1, ORF2(VP1), and ORF3(VP2) (2), with a high incidence of genetic variation (3, 4). In China, circulating FCV strains are branched into genotype I and genotype II based on nucleotide sequences of VP1 (5, 6), and some emerging recombinant FCV strains causing virulent systemic diseases have also been confirmed in recent years (4, 7), resulting in reduced FCV detection rate in a proportion of feline patients. Besides, viral load and viral shedding in tissues are variable among vaccinated and unvaccinated cats (5, 8, 9), which greatly challenges the existing FCV molecular detection methods. Although the real-time PCR method is still the gold standard for FCV nucleic acid detection, the dependence on complicated facilities and skilled personnel limits its application as point of care testing (POCT).

Currently, various orthologs of CRISPR-associated enzymes, including Cas13a nuclease, are applied to establish nucleic acid detection assays with powerful performance, which are introduced as alternative tools for pathogen detection (10, 11). By coupling with recombinase polymerase amplification (RPA) reaction and visual readout, the CRISPR-Cas13a based diagnostic approach has no use for thermal cycler or sophisticated equipment, which makes it feasible as an efficient biosensor in resource-limiting settings. Uniquely, CRISPR-Cas13a exerts its trans-cleavage activity when specific CRISPR RNA (crRNA) binds the target sequence in the ternary complex and shears probe-tagged ssRNA to produce detectable signals (12). By this principle, the SHERLOCK (specific high-sensitivity enzymatic reporter unlocking) platform was developed and exhibited robust RNA nucleic acid detection ability (13) with incomparable advantages compared to the real-time PCR method. It is precisely because of ultrasensitivity, high specificity, and time-saving manipulation that CRISPR-Cas13a based detection assays could be utilized for FCV nucleic acid detection in clinical settings.

This study aims to develop a Cas13a-based visual detection approach for FCV nucleic acid and provides a novel strategy for on-site detection in small animal hospitals or at-home self-testing.

## Materials and Methods

### Protein Expression and Purification

LwCas13a protein was purified as previously described in literature with minor modification (10). Briefly, the plasmid pC013-Twinstrep-SUMO-huLwCas13a (Miaolingbio, China) containing the inserted LwCas13a gene, was transformed into BL21(DE3) Competent E.coli (Tsingke, China). The expression of the target protein was induced with 500 μM (final concentration) IPTG (SigmaAldrich, China) at 25°C for 16 h. The collected bacterial cell pellets were disrupted by an ultrasonic cracker in lysis buffer (20 mM Tris-HCl, 500 mM NaCl, 1%Triton X-100, pH 8.0) with EDTA-free Halt™ Protease Inhibitor Cocktail (ThermoFisher Scientific, USA). The soluble LwCas13a in the supernatant was harvested and filtered through a 0.22 μm filter followed by purification using Ni-NTA gel for affinity chromatography, then removed hybrid proteins by gradient imidazole eluent (50~80 mM) and further eluted with another 500 mM imidazole elution. The fusion LwCas13a protein was eluted with buffer (20 mM Tris-HCl, 500 mM NaCl, 1% Triton X-100, 500 mM imidazole, pH 8.0), then cleaved by 1.5% SUMO protease (Solarbio, China) to remove the SUMO tag. The protein eluate was concentrated by Ultra Centrifugal Filters (Millipore, Germany). Finally, the purified LwCas13a protein was determined by SDS-PAGE and coomassie blue staining, then quantitated using the BCA Protein Assay Kit (ThermoFisher Scientific, USA) and stored at −80°C.

### Preparation of Strains and Clinical Specimens

Positive nucleic acid of *Mycoplasma felis, Chlamydophila felis, Bordetella bronchiseptica* (B.b), feline coronavirus (FCoV) confirmed by real-time PCR were provided by the Medical Diagnostic Laboratory of Veterinary Hosptical of Southwest Minzu University. *Escherichia coli* (E.coli), *Klebsiella Pneumonia* (K.p), Feline herpevirus-1 (FHV-1), feline panleukopenia (FPV) isolates, and feline calicivirus (FCV) strains (SMU-F4-2020, SMU-B5-2020, and SMU-B22-2020) were provided by Department of Clinical Veterinary Medicine of Southwest Minzu University. Conjunctival, nasal, or oropharyngeal swabs were collected from 56 cats with upper respiratory symptoms who visited the Veterinary Hospital of Southwest Minzu University in Chengdu city, Sichuan province from 2020 to 2021. The nucleic acids were extracted by DNA/RNA extract kit or bacterial genomic extraction kit (Tiangen Biotech, China), and all nucleic acid RNA were reversely transcribed into cDNA with PrimeScript^TM^ RT reagent Kit (Takara, China).

### Design of Primers, Probes, and CrRNA Preparation

**Figure 1:**
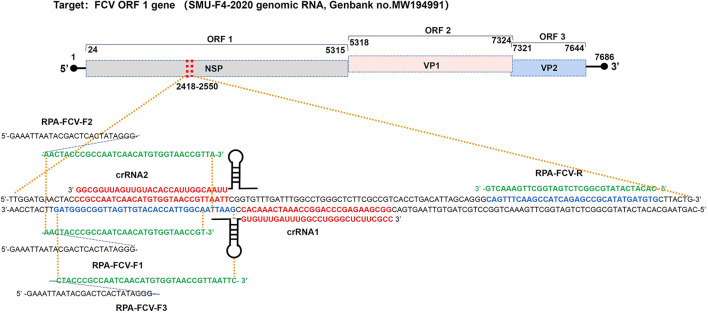
Binding sites of RPA primers and crRNA oligonucleotides targeting to ORF-1 gene within the FCV genome map. crRNA1, crRNA2, and RPA primers were designed in positive and negative strands of the FCV ORF1 gene (from 2418-2550 nt), respectively, according to the conserved sequence of epidemic FCV strain SMU-F4-2020 from Southwest China.

The highly conserved sequences of the feline calicivirus ORF-1 gene were selected by aligning the full-length FCV genomic RNA from GenBank (https://www.ncbi.nlm.nih.gov/) to design specific RPA primers containing T7 promoter by Prime Primer 6.0 software and BLAST (https://blast.ncbi.nlm.nih.gov/). The pGM-T (Amp) plasmid containing 133 bp FCV ORF-1 gene sequence (from 2418-2550 nt) from FCV strain SMU-F4-2020 (Genbank no. MW194991) were constructed using pGM-T Ligation Kit (TIANGEN Biotech, China). The designed primers, probes, and crRNA oligonucleotides were synthesized by Sangon Biotech, China. The crRNA oligonucleotides containing T7 promoter were annealed into dsDNA using an annealing buffer for DNA oligos (Beyotime, China) and HiScribe T7 RNA synthesis kit (NEB, China). The synthetic crRNA was digested with DNase I (NEB, China) at 37°C for 15 min and purified with Monarch RNA Purification Kit (NEB, China) according to the instruction manual. The concentration of synthetic crRNA was determined using a Qubit 3.0 fluorometer (ThermoFisher Scientific, USA). All sequences used in this study were shown in Supplementary Table 1. The primer binding sites were shown in Figure 1.

### Establishment of FCV Cas13a Assays

The FCV-Cas13a assays combined RPA reaction and LwCas13a trans-cleavage system containing T7 transcription. For RPA, 1 μl cDNA or DNA was amplified in a 50 μl reaction system for 20 min at 39°C, according to the instruction of the TwistAmp® Basic kit (TwistDx, UK). The optimal RPA primer pairs were determined by quantitation of PRA amplicons with a Qubit 3.0 fluorometer (Thermofish Scientific, USA). The 30 μl FCV-Cas13a reaction system that consisted of 1 μl RPA amplicons, 0.5~100 nM Cas13a, crRNA (450 nM crRNA1, 450 nM crRNA2 or dual crRNAs, respectively), 1.6 U/μl RNase inhibitor (NEB, USA), 0.2 pM FD-reporter or 60~360 nM FQ-reporter, 3 μl T7 RNA Polymerase (NEB, China), 3 μl RNAPol Reaction Buffer, 1.5 μl NTP Buffer Mix and supplementary reaction buffer (60 mM NaCl, 40 mM Tris-HCl, 6 mM MgCl_2_, pH 7.3) was incubated for 60 min at 37°C.

To evaluate the validity of FCV-Cas13a assays, seven reaction systems (RSs) consisting of different components were tested. NTP Buffer mix and reaction buffer were all fixed, and other essential components were deleted in turn. crRNAs containing each 450 nM crRNA1 and crRNA2 were added into RSs.

For immunochromatographic detection (FCV-Cas13a-LFD), HybriDetect Dipstick (Twist Dx, UK) was dipped into a reaction buffer with 20 μl FCV-Cas13a products and 100 μl hybridetect assay buffer and incubated for 5 min for readout. For fluorescence detection (FCV-Cas13a-FLUOR), the reaction tubes with FQ-reporter were put under the LED blue light illuminator (BluPAD, Bio-Helix) (see Supplementary Figure 1) or the Bio-Rad ChemiDoc MP imaging system with its built-in UV channel. The fluorescence signals were collected within 75 min in Quantstudio 3 Real-Time PCR System (ThermoFisher Scientific, USA). To determine the validity of the FCV-Cas13a assay, seven reaction systems with different components were tested. The performance of crRNA1, crRNA2, and duplex crRNAs (crRNA1/2) was verified by FCV-Cas13a-FLUOR. Besides, the optimal concentration of Cas13a and FQ-reporter were also evaluated by FCV-Cas13a-FLUOR.

### Sensitivity and Specificity of FCV-Cas13a Assays

For sensitivity testing, the serially diluted plasmid DNA templates (10^10^~10^−2^ copies/μl) were prepared and detected by the two-step FCV-Cas13a assays and SYBR Green RT-qPCR (14). The plasmid DNA copy number was determined using the following formula:{[6.02 × 10^23^ × dsDNA concentration (ng/μl) ×10^−9^]}/[DNA in length ×660]. The specificity of FCV-Cas13a assays was verified by detecting nucleic acids of feline associated pathogens (*Mycoplasma felis, Chlamydophila felis*, B.b, FCoV, *E.coli*, K.p, FHV-1, FPV, and FCV). All specimens with a Ct value of ≤ 35 were considered positive by the RT-qPCR test.

### Comparison of FCV-Cas13a Assays and RT-qPCR for Field Detection

Extracted nucleic acids from 56 clinical specimens were detected using FCV-Cas13a assays and RT-qPCR method (14), respectively, and the comparative specificity and sensitivity of the two methods were statistically analyzed.

### Image and Statistical Data Analysis

Immunochromatographic and fluorescence readouts were imaged by camera and automatically adjusted using Image J software. The endpoint raw fluorescence was determined by the CFX Real-time PCR System (Bio-Rad, USA). All data in this study for drawing fluorescence plottings were analyzed using GraphPad Prism 8 software and *t*-test. The comparison between FCV-Cas13a assays and the RT-qPCR method was analyzed using SPSS 16.0 software and the Chi-square test.

## Results

### LwCas13a Expression and Purification

**Figure 2:**
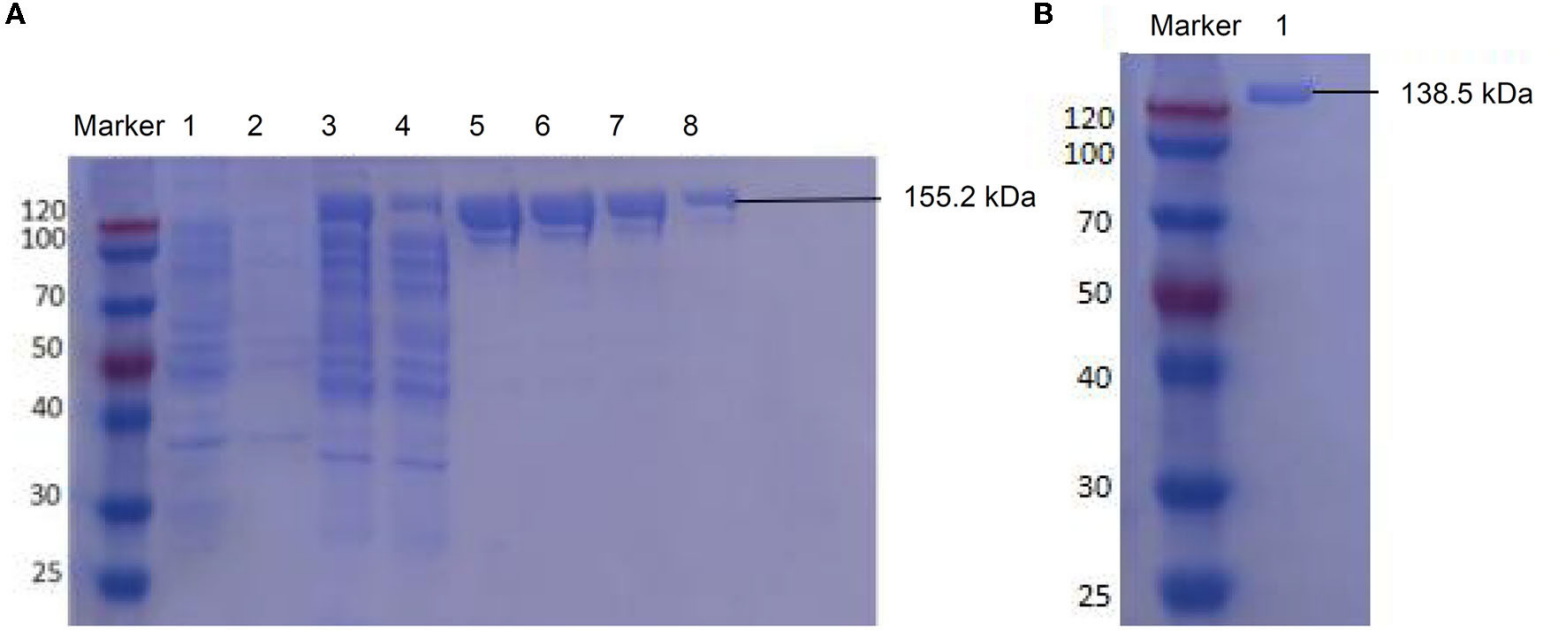
SDS-PAGE electrophoresis for LwCas13a purification. **(A)** Undigested LwCas13a protein (155.2 kDa). lane M: protein marker, lane 1: uninduced bacterial sample, lane 2: precipitation of induced sample, lane 3: supernatant of the induced sample, lane 4: flow-through sample, lane 5–8: elution with 500 mM imidazole. **(B)** Digested LwCas13a protein (138.5 kDa). lane marker: protein marker, lane 1: the purified LwCas13a protein.

The LwCas13a protein expression was induced for 16 h at 25°C with 500 μM IPTG. The harvested pellets were resuspended, lysed, and concentrated. The expressed 155.2 kDa LwCas13a with tag protein was mainly dissoved in the supernatant and eluted by elution buffer (Figure 2A), then digested using SUMO protease to remove the SUMO tag. The purified 138.5 kDa LwCas13a protein was confirmed by Coomassie staining (Figure 2B) and the initial concentration was 1605.1 ng/μl.

### Screening of the Optimal RPA Primers

**Figure 3:**
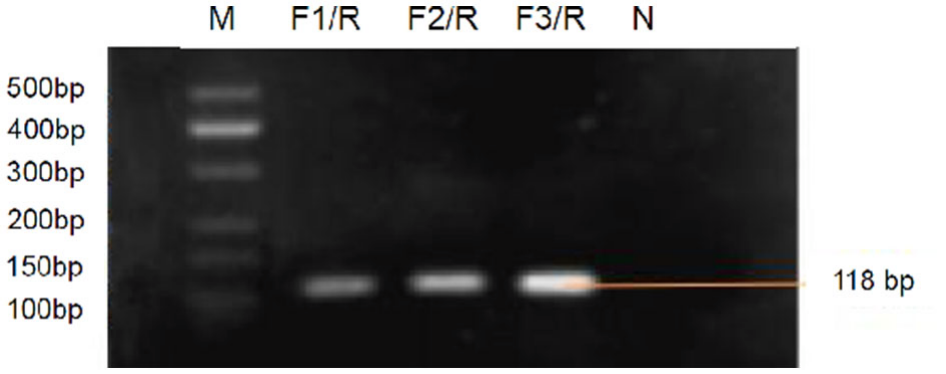
Gel electrophoresis for RPA products amplified by F1/R, F2/R, and F3/R primers, respectively lane M, standard molecular weight. lane *N*, Negative control (RNase-Free distilled water).

Three genomic RNA of FCV strains (SMU-F4-2020, SMU-B5-2020, and SMU-B22-2020) from Southwest China and 15 FCV strains on NCBI were aligned showing different mutant sites on the target gene sequence (see Supplementary Figure 2). Target gene fragments of RPA primers and RT-qPCR were overlapped, however, RT-qPCR forward primer covered more mutant sites than RPA forward primer, which possibly indicated reduced amplification efficiency for RT-qPCR. The purified RPA amplicons (approximate 118 bp) were determined by 2% gel electrophoresis (Figure 3). The nucleic acid concentration of RPA products by F3/R primers (106.3 ng/μl) was higher than that of F1/R (50.4 ng/μl) and F2/R (66.7 ng/μl) primers, respectively, which suggested that F3/R primers were optimal for later RPA reaction.

### Validity of FCV-Cas13a Assays

**Figure 4:**
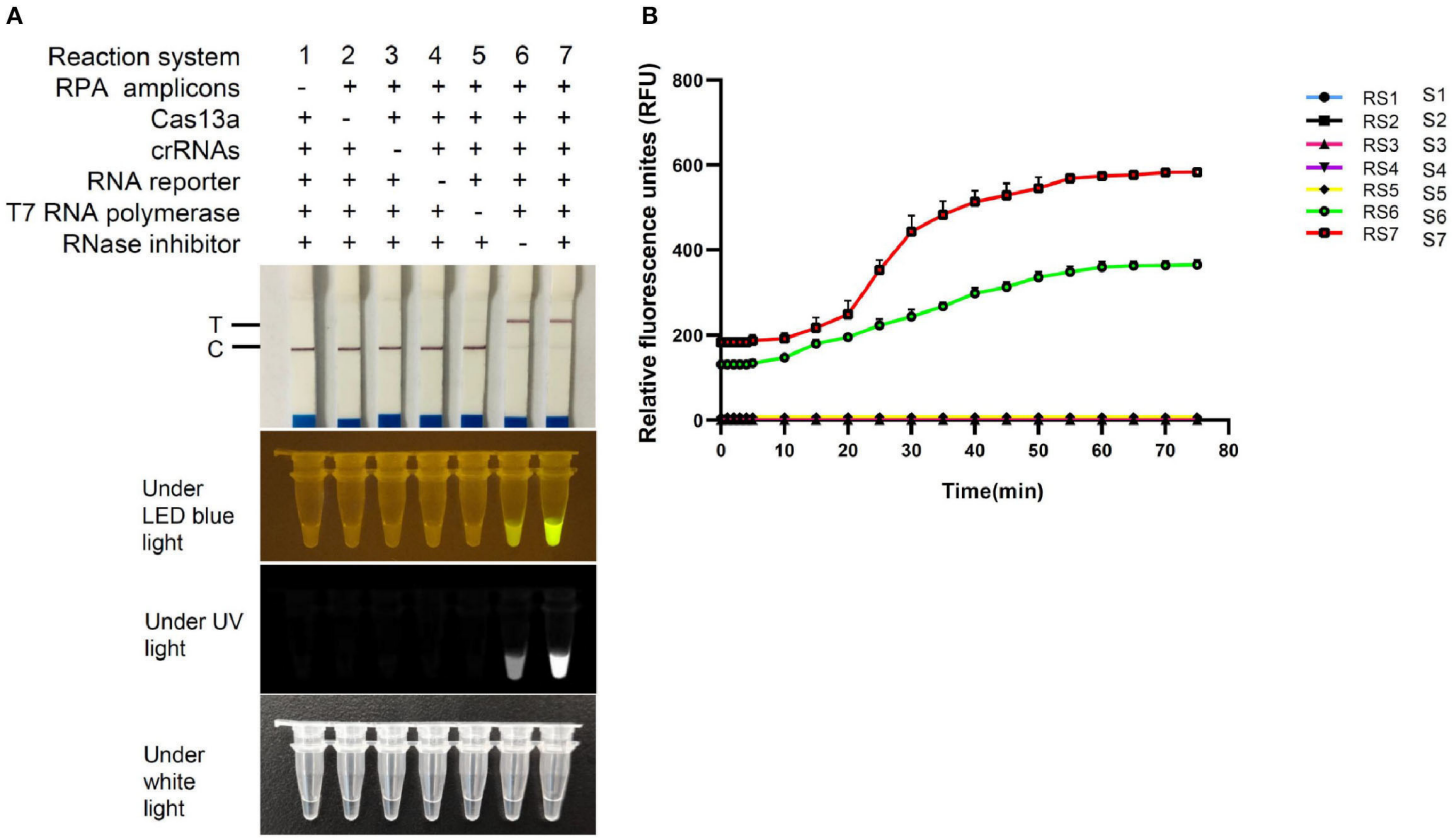
Validation of FCV-Cas13a assays. **(A)** Detection results of FCV-Cas13a-LFD and FCV-Cas13a-FLOUR. Visual readouts of seven RSs with different components was recorded by endpoint imaging of lateral flow dipstick or fluorescence detector. **(B)** Fluorescence plottings of seven RSs by FCV-Cas13a-FLUOR. Both RS6 and RS7 were valid, but RS7, by contrast, was more robust. Each experiment was repeated three times.

To determine the validity of FCV-Cas13a assays, seven reaction systems (Figure 4A) with different components were tested. The absence of arbitrary components in the reaction system (RS) made the assays invalid, except for the RNase inhibitor, which merely resulted in attenuated fluorescence signals (Figures 4A,B). The Cas13a nuclease and RNA-FQ reporter were crucial determinants to fluorescence readout, therefore the optimal amount of the two components for saturated fluorescence was studied by fixing the concentration of the other components. The optimal concentration of Cas13a and RNA-FQ reporter were 50 and 240 nM, respectively, as shown in Supplementary Figures 3, 4. Besides, the strongest fluorescence signals were obtained in crRNA1/2 RS compared to crRNA1 and crRNA2 RS, respectively, in a plasmid DNA concentration-dependent manner as shown in Supplementary Figure 5. The final version of 30 μl RS consisted of 1 μl RPA amplicons, 50 nM Cas13a, 450 nM crRNA1/2, 1.6 U/μl RNase inhibitor, 240 nM FQ-reporter or 0.2 pM FD-reporter, 3 μl T7 RNA Polymerase, 3 μl RNAPol Reaction Buffer, 1.5 μl NTP Buffer Mix, and reaction buffer was added to 30 μl. The visual readout could be observed within 30 min at 37°C.

### Specificity and Sensitivity

**Figure 5:**
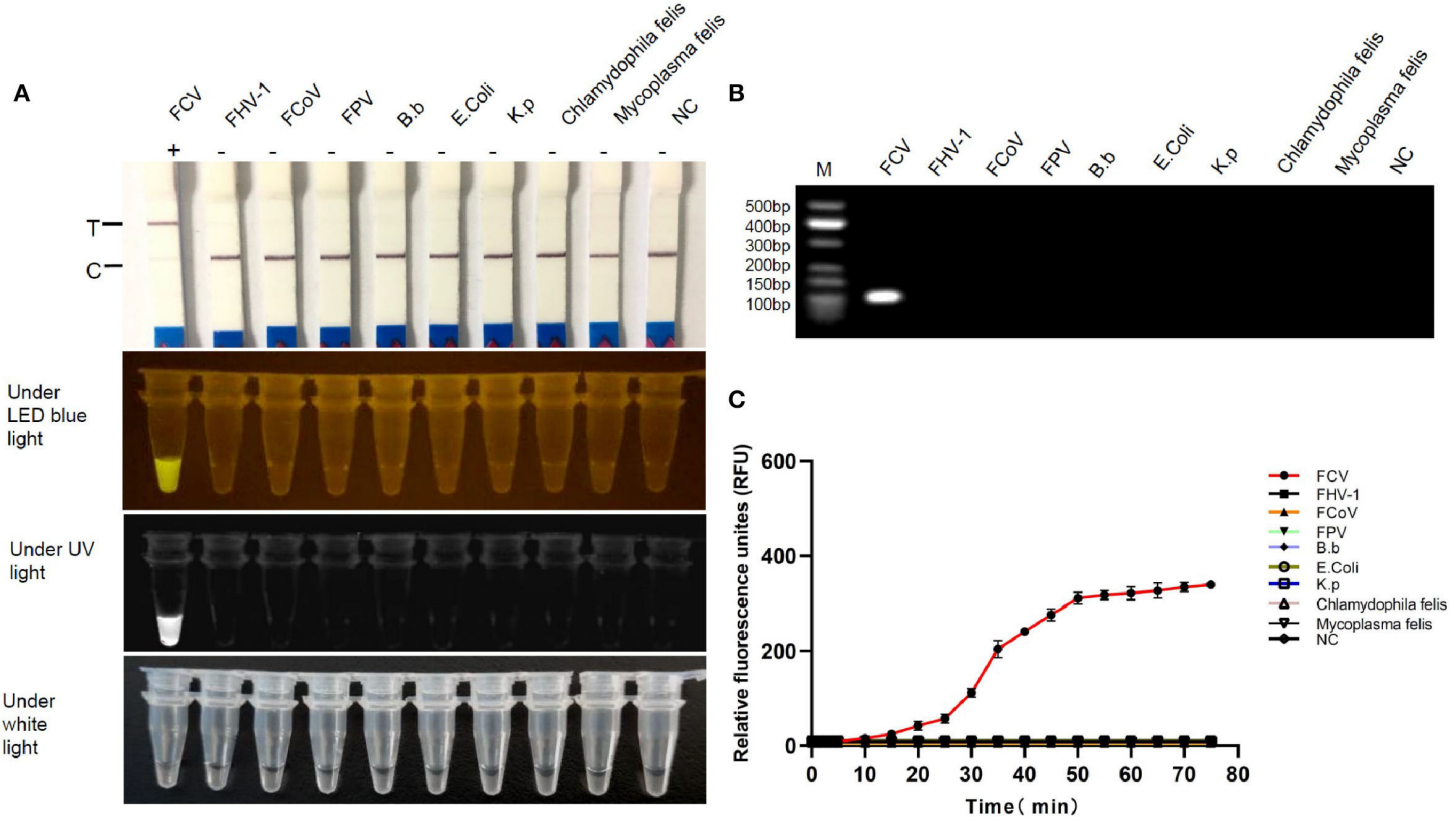
Specificity testing of FCV-Cas13a assays. **(A)** Detection results of FCV-Cas13a-LFD and FCV-Cas13a-FLUOR. Both methods only detected FCV RNA but no other pathogens. **(B)** Electrophoresis of RPA amplicons of all tested pathogens. Lane M, standard molecular weight; lane NC, negative control (RNase-Free distilled water). **(C)** Detection results by FCV-Cas13a-FLUOR were shown in fluorescence plottings. Each experiment was repeated three times.

**Figure 6:**
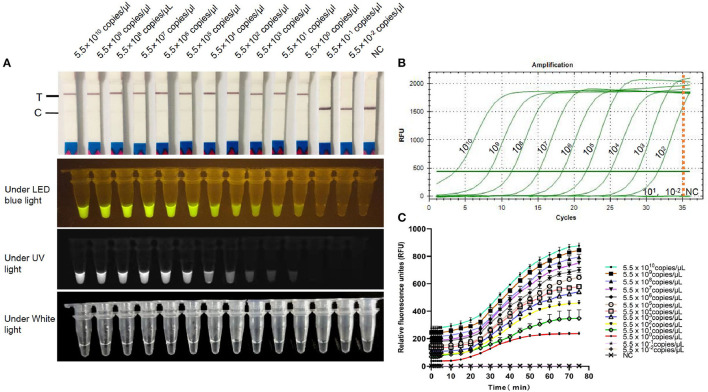
Sensitivity testing of FCV-Cas13a assays. **(A)** Detection results of FCV-Cas13a-LFD and FCV-Cas13a-FLUOR. Gradiently diluted FCV plasmid DNAs were detected using FCV-Cas13a assays. The minimum detection limit was 5.5 copies/μl. **(B)** RT-qPCR amplification curves. No amplification curve was recorded when the plasmid DNA concentration was lower than 5.5 ×10^2^ copies/μl. **(C)** Detection results by FCV-Cas13a-FLUOR were shown in fluorescence profiles. Each experiment was repeated three times. NC, Negative control (RNase-Free distilled water).

FCV-Cas13a assays only detected FCV nucleic acid, while no cross-reaction with other feline pathogens (Figure 5). Besides, serial dilution of FCV plasmid DNAs were added into FCV-Cas13a RSs, respectively, for 60 min incubation. The initial concentration of plasmid DNA was 79.2 ng/μl and the converted copy number was 5.5 ×10^11^ copies/μl. The minimum detection limit was 5.5 copies/μl for both FCV-Cas13a-LFD and FCV-Cas13a-FLUOR, which were far more sensitive than RT-qPCR (5.5 ×10^2^ copies/μl) (Figure 6).

### Clinical Detection and in Comparison With RT-qPCR

**Figure 7:**
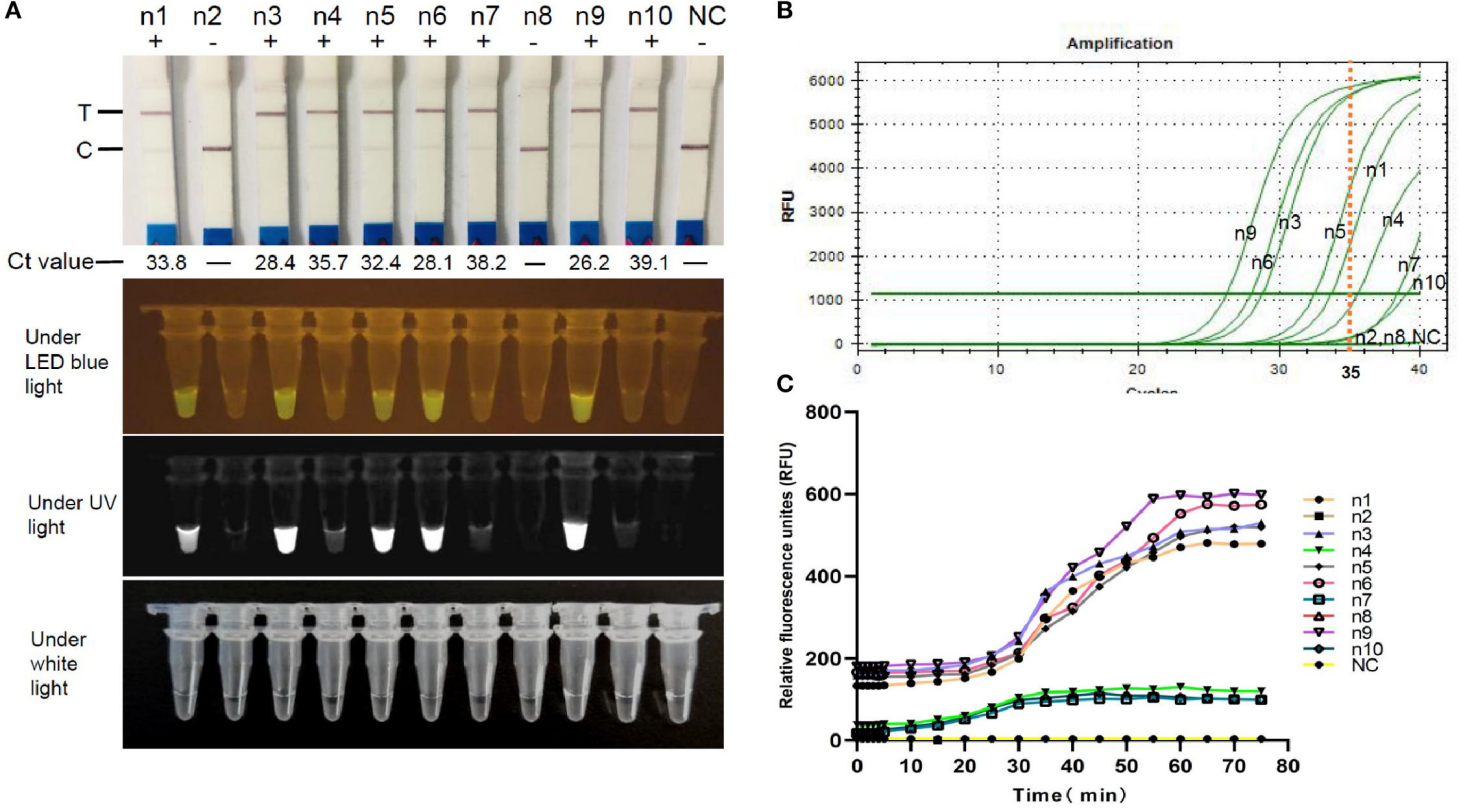
Field detection using FCV-Cas13a assays. **(A)** Detection results of 10 representative specimens by FCV-Cas13a-LFD and FCV-Cas13a-FLUOR. **(B)** Detection results by RT-qPCR. **(C)** Detection results by FCV-Cas13a-FLUOR were shown in fluorescence profiles. NC, Negative control (RNase-Free distilled water).

To confirm the validity of FCV-Cas13a assays in clinical settings, 10 representative specimens were successfully tested. The detection rate of both FCV-Cas13a assays (Figures 7A,C) were consistent, but visual results of FCV-Cas13a-LFD were easier to be interpreted than that of FCV-Cas13a-FLUOR (n4, n7, n10) in specimens with extremely low copies of FCV nucleic acids (Figure 7A). In addition, more positive specimens were detected by FCV-Cas13a assays (8/10) than by RT-qPCR (5/10) (Figures 7B,C). Three presumptive positive specimens by RT-qPCR (n4, n7, and n10, Ct value >35) were confirmed as positive by FCV-Cas13a assays and DNA sequencing. Besides, a similar result was also shown in the expanded 56 clinical specimens. The detection rate of FCV-Cas13a assays (FCV-Cas13a-LFD and FCV-Cas13a-FLOUR) was 67.9% (38/56), which is significantly higher than that of RT-qPCR (25/56, 44.6%) (*p* < 0.001) (see Supplementary Figures 6–8). FCV-Cas13a assays showed good consistency with RT-qPCR in positive specimens but were more reliable for the detection of presumptive positive specimens (Table 1).

**Table 1:** Comparison between FCV-Cas13a assays and RT-qPCR method used for detecting 56 clinical specimens.

		**RT-qPCR**		**Total**	**Sensitivity (%) (95% CI)**	**Specificity (%) (95% CI)**
		**+**	**–**			
FCV-Cas13a assays	+	25	13	38	100	58.1%
	–	0	18	18		
Total		25	31	56		

## Discussion

The newly emerging FCV variants are prevalent in Southwest China and have been reported as an increased onset risk to native feline patients (5). Early diagnosis of these infectious agents is crucial to prevent the transmission of this intractable virus in cat populations. Various RT-qPCR methods for FCV detection had been reported (15, 16), but multiple mutations in target genes of epidemic FCV variants could result in a mismatch between amplification primers and target sequences and subsequently reduced positive detection rate in clinical settings. It is well-known that symptoms of FCV infection, to an extent, are non-specific and similar to other upper respiratory pathogens (e.g., FHV-1 and *Chlamydia felis*). Therefore, an accurate and rapid diagnosis of these co-infected pathogens is crucial to early-stage disease prevention and management.

This study developed two Cas13a-based visual assays (FCV-Cas13a-LFD and FCV-Cas13a-FLUOR) for FCV detection with high efficiency without cross-reaction with other associated pathogens. The assays were more reliable than the reference RT-qPCR method and had a lower minimum detection limit than a recently reported Taqman real-time PCR method (minimum detection limit was 50 copies/μl) (17). The high sensitivity and specificity of FCV-Cas13a assays may be attributed to the integration of specific RPA as a signal amplifier, duplex crRNAs, and Cas13a-activated cascading fluorescence signals. The high efficiency of RPA reaction was essential for FCV-Cas13a assays, which could rapidly accumulate nucleic acid sequences at low constant temperature (35~42°C) and provided abundant targets for crRNAs to recognize and bind with. In a recent report, one RPA variant (ERA) was applied to detect FCV variants with a lower detection limit (3.2 TCID_50_) with lateral flow dipstick readout (18), which highlighted the high-performance of PRA for FCV detection. However, lower amplification temperature would enable RPA primers to form dimers or match non-target sites, thereby generating non-specific products and false positive results (19). For this reason, the application of crRNA-guided CRISPR-Cas13a trans-cleavage activity would further enhance the specificity of RPA detection. Because of the high genetic diversity of co-circulating FCV strains in cat populations (5, 7), the introduction of unique dual crRNAs into RSs could minimize the possible mismatch between guide RNA and target sequences to validate the detection system (20). To this end, we applied FCV-Cas13a assays to detect FCV circulating in Southwest China, and obtained consistent test results for positive specimens with the RT-qPCR method but a higher detection rate for presumptive positive specimens.

To our knowledge, the FCV-Cas13a assays that target the FCV ORF-1 gene are firstly established by integrating RPA with the CRISPR-Cas13a detection system, and the test results can be directly interpreted by the naked eye. The FCV-Cas13a-FLUOR method obtains real-time readout by collecting gradually enhanced detection fluorescence signals over time, and could realize one-tube detection (concurrent RPA and Cas13a tans-cleavage reaction) with contamination-free readout (20), but with reduced detection rate compared to two-step detection systems (21). Additionally, the optical readout is ambiguous for FCV-Cas13a-FLUOR in specimens containing ultra-low copies of nucleic acid. In a sense, FCV-Cas13a-LFD is an instrument-free visual detection method, which may be more suitable for field detection in resource-poor settings. However, the high risk of environmental FCV contamination for the open-lid FCV-Cas13a-LFD method would limit its in-house application, especially in space-restricted clinics. Hence, a contamination-free and insulated LFD kit should be designed wisely for clinical use.

Since the FCV-Cas13a assays generate readout at the endpoint, it is convenient to record and report the results promptly by smartphone (12), achieving a remote diagnosis for self-testing pet owners. Although the estimated cost of these assays is reasonably high and acceptable for small animal hospitals, it can be significantly decreased when specialized for large-scale production. Therefore, the FCV-Cas13a assays would be a versatile and reliable point of care method for FCV RNA detection in small animal hospitals or self-testing at home.

## Conclusion

FCV-Cas13a assays provided a robust and visual approach for FCV nucleic acids detection and could help with point of care diagnosis and monitoring of epidemic FCV in clinical practice.

## Data Availability Statement

The original contributions presented in the study are included in the article/Supplementary Material, further inquiries can be directed to the corresponding authors.

## Author Contributions

JH: study design, methodology establishment, data sorting, manuscript writing, and revision. YLiu: methodology establishment, data sorting, and writing—original draft. YH: methodology establishment, data sorting, and analysis. XY: collaborated in the writing, analysis, and interpretation of data. YLi: study design, analysis, funding acquisition, and writing—review and editing. All authors contributed to the article and approved the submitted version.

## Funding

This study was supported by the Fundamental Research Funds for the Central Universities, Southwest Minzu University (Grant No. 2020NQN32) and Start-up Scientific Research Foundation for Talent Introduction, Southwest Minzu University (Grant No. RQD2021098).

## Conflict of Interest

The authors declare that the research was conducted in the absence of any commercial or financial relationships that could be construed as a potential conflict of interest.

## Publisher's Note

All claims expressed in this article are solely those of the authors and do not necessarily represent those of their affiliated organizations, or those of the publisher, the editors and the reviewers. Any product that may be evaluated in this article, or claim that may be made by its manufacturer, is not guaranteed or endorsed by the publisher.
