# Supplementary material for: CRISPR-Cas13a Based Visual Detection Assays for Feline Calicivirus Circulating in Southwest China
Source: Front Vet Sci. 2022 Jul 11;9:913780. doi: 10.3389/fvets.2022.913780 (PMC9310557; doi:10.3389/fvets.2022.913780)
Supplement: Supplementary file 1 [file Table_1.pdf]

Supplementary Material

Supplementary Table 1. Primers and oligonucleotides for FCV-Cas13a assays.

**Primers**	**Sequence**	**Size**	**Source**
RT-PCR-FCV-F	5'-TTGGATGAACTACCCGCCA-3'	133 bp	This study
RT-PCR-FCV-R	5'-CAGTAAGCAC ATCATATGC-3'		
RT-qPCR-FCV-F	5'-TAATTCGGTGTTTGATTTGGCCTGGGCT-3'	83 bp	[15]
RT-qPCR-FCV-R	5'-CATATGCGGCTCTGATGGCTTGAAACTG-3'		
T7-crRNA-F	5'-GAAATTAATACGACTCACTATAGGG-3'	93 bp	this study
T7-crRNA-1-R	5'-GGCGAAGAGCCCAGGCCAAATCAAACACGTTTTAGTCCCCTTCG TTTTTGGGGTAGTCTAAATCCCCTATAGTGAGTCGTATTAATTTC-3'		
T7-crRNA-2-R	5'-CCGCCAATCAACATGTGGTAACCGTTAAGTTTTAGTCCCCTTCGT TTTTGGGGTAGTCTAAATCCCCTATAGTGAGTCGTATTAATTTC3'		
RPA-FCV-F1	5'-GAAATTAATACGACTCACTATAGGGAACTACCCGCCAATCAACA TGTGGTAACCGT-3'	118bp	This study
RPA-FCV-F2	5'-GAAATTAATACGACTCACTATAGGGAACTACCCGCCAATCAACA TGTGGTAACCGTTA-3'		
RPA-FCV-F3	5'-GAAATTAATACGACTCACTATAGGGCTACCCGCCAATCAACATG TGGTAACCGTTAATTC-3'		
RPA-FCV-R	5'-CACATCATATGCGGCTCTGATGGCTTGAAACTG-3'		
FD-reporter	5'-6-FAM-UUUUUUUUUUUUUU-Digoxigenin-3'		
FQ-reporter	5'-6-FAM-UUUUUUUUUUUUUU-BHQ1-3'		
