# Supplementary material for: CRISPR-Cas13a Based Visual Detection Assays for Feline Calicivirus Circulating in Southwest China
Source: Front Vet Sci. 2022 Jul 11;9:913780. doi: 10.3389/fvets.2022.913780 (PMC9310557; doi:10.3389/fvets.2022.913780)
Supplement: Supplementary file 2 [file Data_Sheet_1.pdf]

Supplementary Material

Supplementary Table 1. Primers and oligonucleotides for FCV-Cas13a assays.

Primers	Sequence	Size	Source
RT-PCR-FCV-F	5'-TTGGATGAACTACCCGCCA-3'	133 bp	This study
RT-PCR-FCV-R	5'-CAGTAAGCAC ATCATATGC-3'		
RT-qPCR-FCV-F	5'-TAATTCGGTGTTTGATTTGGCCTGGGCT-3'	83 bp	[14]
RT-qPCR-FCV-R	5'-CATATGCGGCTCTGATGGCTTGAAACTG-3'		
T7-crRNA-F	5'-GAAATTAATACGACTCACTATAGGG-3'	93 bp	this study
T7-crRNA-1-R	5'-GGCGAAGAGCCCAGGCCAAATCAAACACGTTTTAGTCCCCTTCG TTTTTGGGGTAGTCTAAATCCCCTATAGTGAGTCGTATTAATTTC-3'		
T7-crRNA-2-R	5'-CCGCCAATCAACATGTGGTAACCGTTAAGTTTTAGTCCCCTTCGT TTTTGGGGTAGTCTAAATCCCCTATAGTGAGTCGTATTAATTTC3'		
RPA-FCV-F1	5'-GAAATTAATACGACTCACTATAGGGAACTACCCGCCAATCAACA TGTGGTAACCGT-3'	119bp	This study
RPA-FCV-F2	5'-GAAATTAATACGACTCACTATAGGGAACTACCCGCCAATCAACA TGTGGTAACCGTTA-3'	119bp	
RPA-FCV-F3	5'-GAAATTAATACGACTCACTATAGGGCTACCCGCCAATCAACATG TGGTAACCGTTAATTC-3'	117bp	
RPA-FCV-R	5'-CACATCATATGCGGCTCTGATGGCTTGAAACTG-3'		
FD-reporter	5'-6-FAM-UUUUUUUUUUUUUU-Digoxigenin-3'		
FQ-reporter	5'-6-FAM-UUUUUUUUUUUUUU-BHQ1-3'		

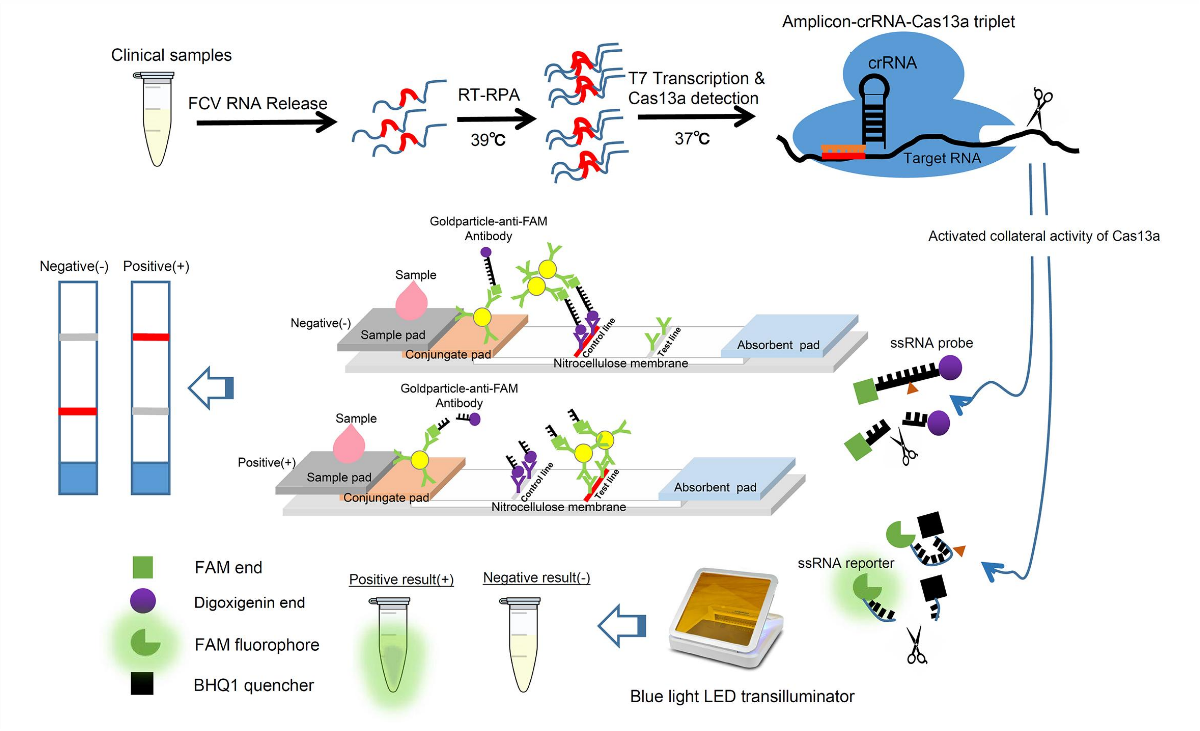

Supplementary Figure 1. Workflow of FCV-Cas13a assays (FCV-Cas13a-LFD and FCV-Cas13a-FLUOR).

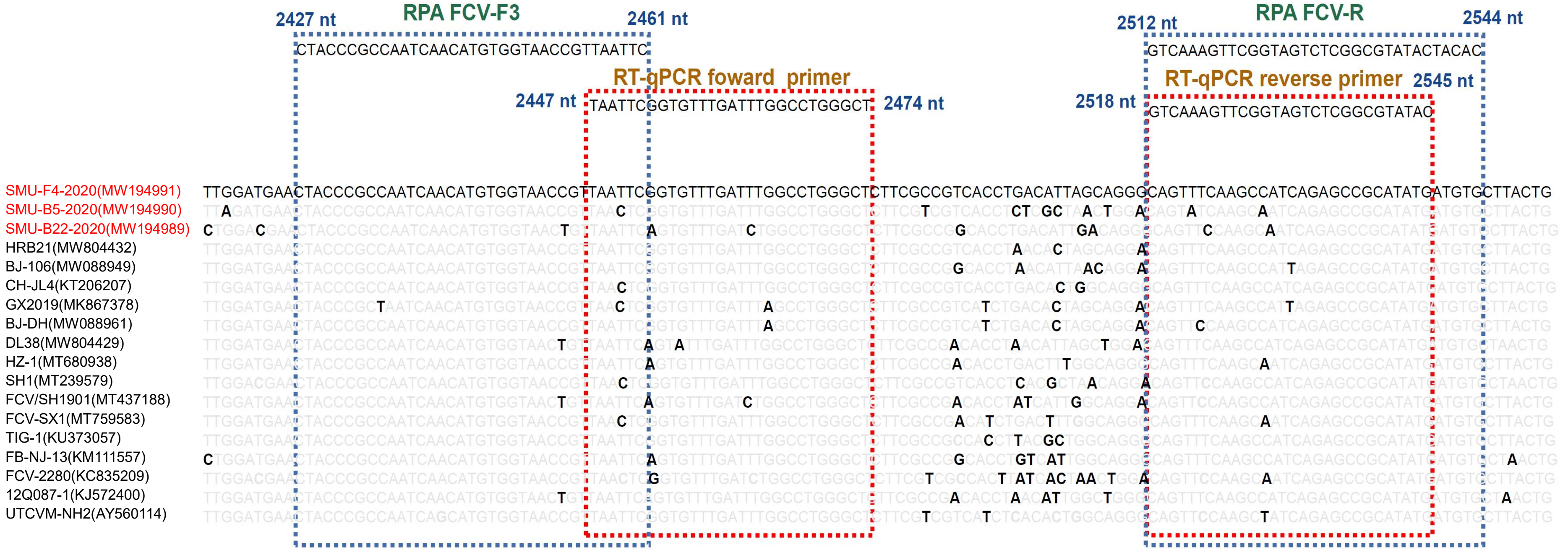

Supplementary Figure 2. RPA primers' binding sites and target gene sequence analysis among different FCV strains. Target gene fragments recognized by RPA primers (RPA-FCV-F3/R) and RT-qPCR primers[14] were overlapped, however RT-qPCR forward primer covered more mutation sites.

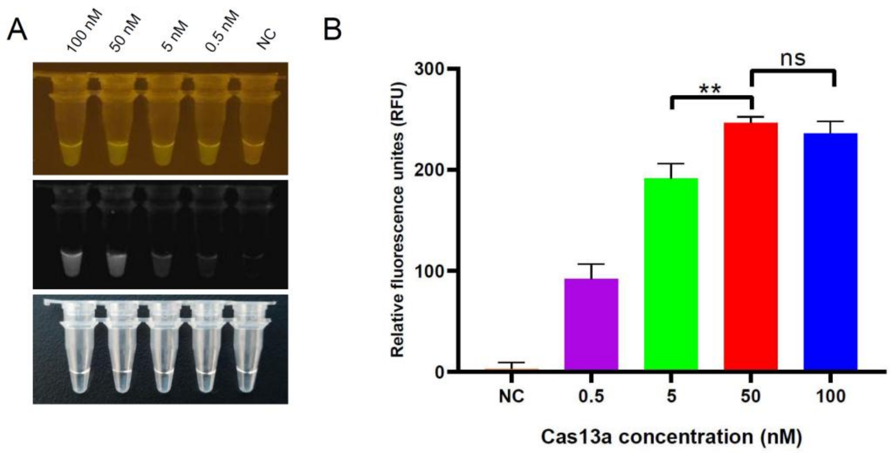

Supplementary Figure 3. (A) Screen optimal concentration of Cas13a in RSs with low concentration of plasmid DNA (55 copies/μl) using FCV-Cas13a-FLUOR. **(B)** Endpoint fluorescence intensity of RSs with tested concentrations of Cas13a. Each experiment was repeated three times. NC, negative control (RNase-Free distilled water) ; ** $p < 0.01$; ns, not significant.

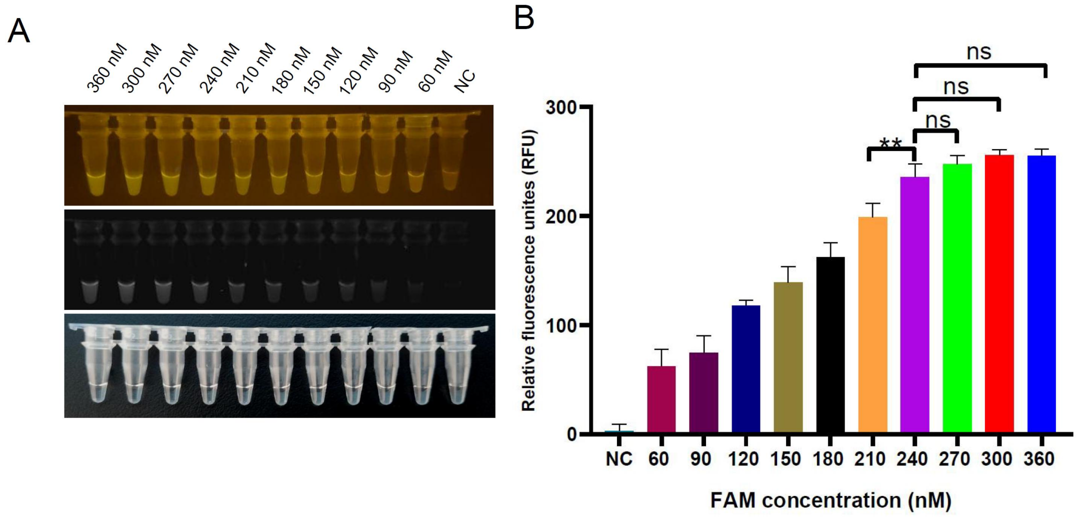

Supplementary Figure 4. (A) Screening optimal concentration of FAM reporter in RSs with low concentration of plasmid DNA (55 copies/μl) using FCV-Cas13a-FLUOR. **(B)** Endpoint fluorescence intensity of RSs with tested concentrations of FAM reporter. Each experiment was repeated three times. NC, negative control (RNase-Free distilled water) ; ** $p < 0.01$; ns, not significant.

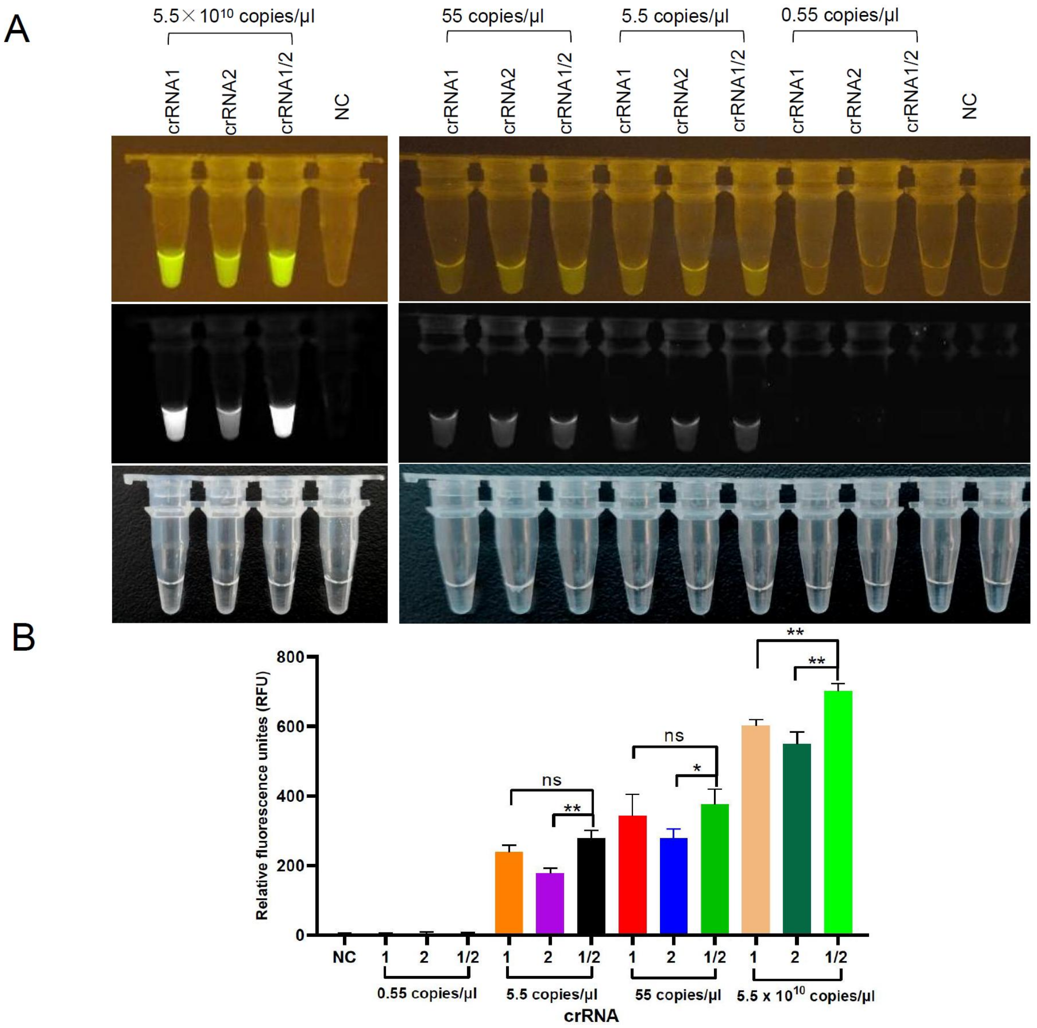

Supplementary Figure 5. (A) The testing of crRNAs efficiency using FCV-Cas13a-FLUOR in RSs with decreaing plasmid DNA concentration. The activity of dual crRNAs (crRNA1/2) was more robust than that of crRNA1 and crRNA2 respectively, but was in plasmid DNA concentration-dependent manner. **(B)** Endpoint fluorescence intensity of RSs with tested crRNAs. Each experiment was repeated three times. NC, negative control (RNase-Free distilled water) ; * $p < 0.05$; ** $p < 0.01$; ns, not significant.

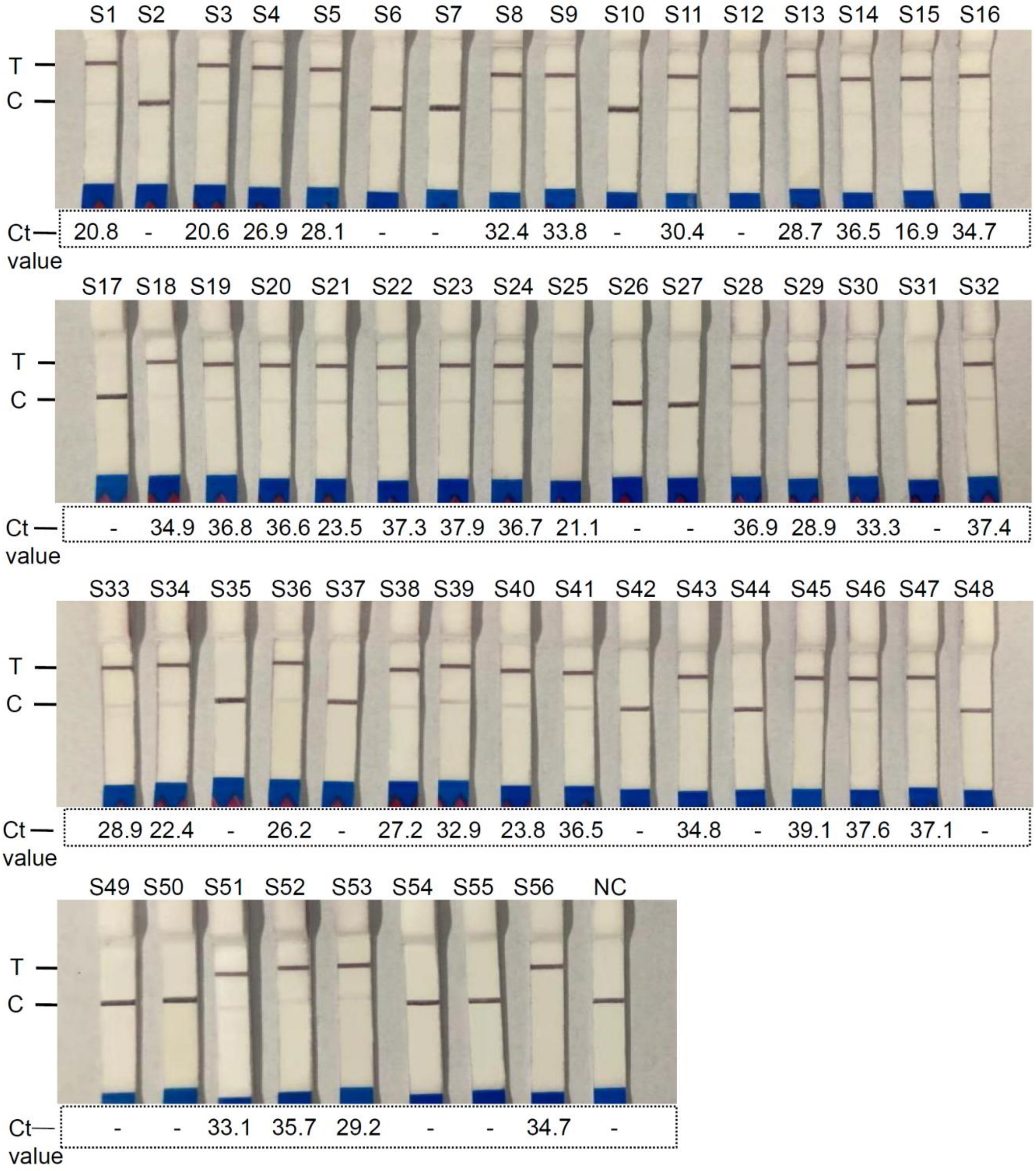

Supplementary Figure 6. Visual detection of 56 clinical specimens (S1-56) using FCV-Cas13a-LFD. Ct values for each sample were shown under the dipsticks respectively. NC, negative control (RNase-Free distilled water).

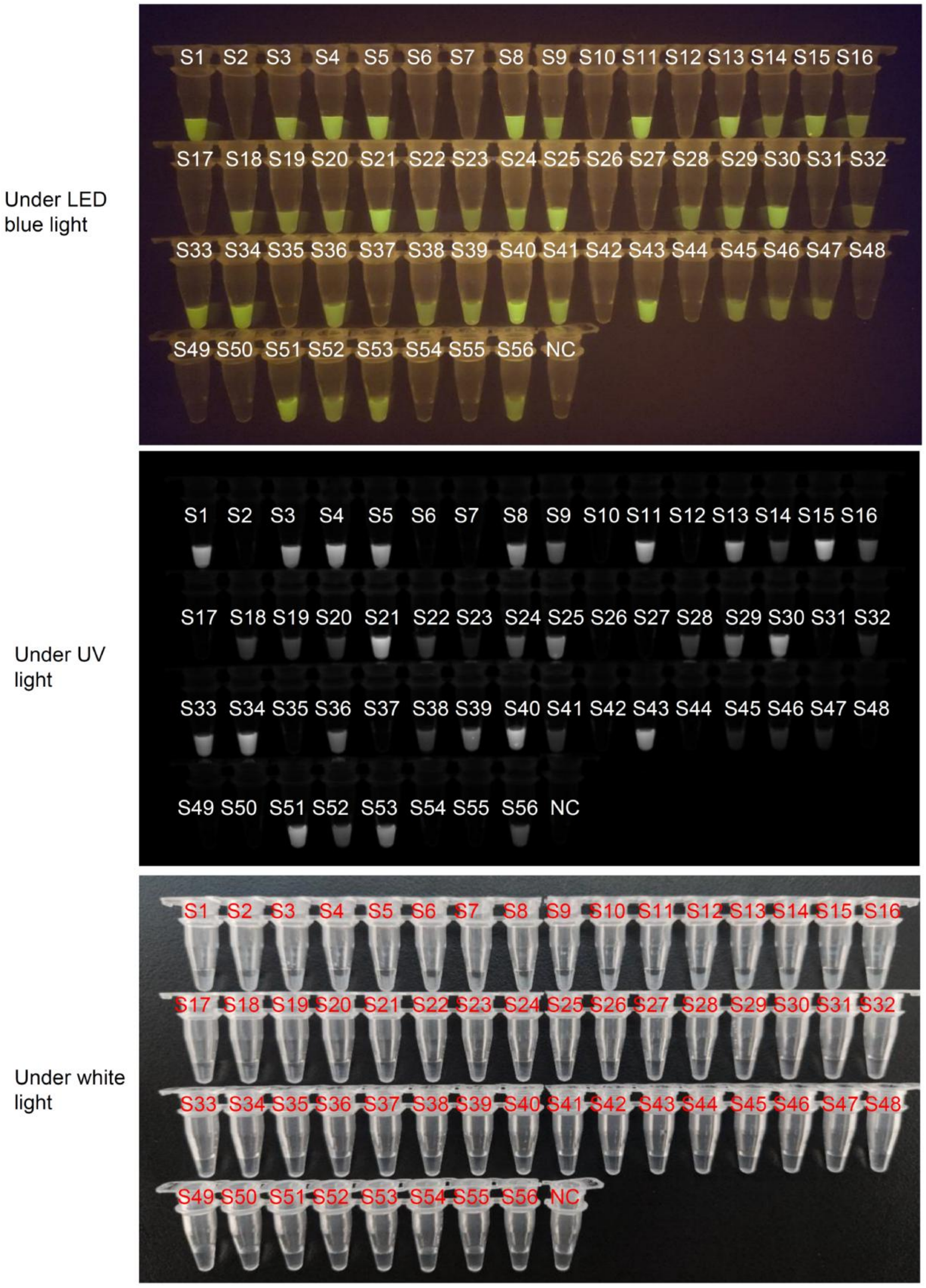

Supplementary Figure 7. Visual detection of 56 clinical specimens (S1-56) using FCV-Cas13a-FLUOR. NC, negative control (RNase-Free distilled water).

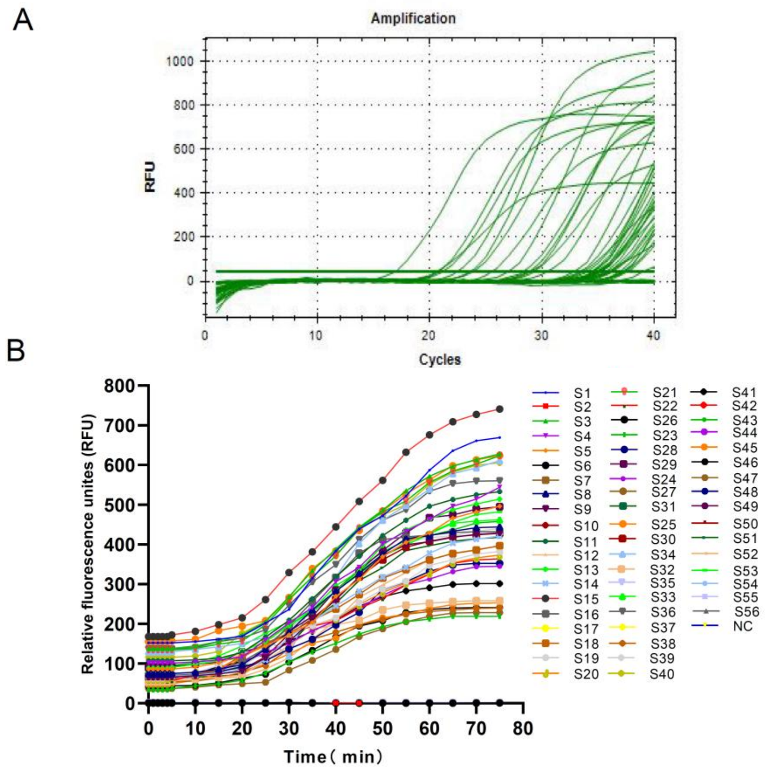

Supplementary Figure 8. (A) Amplification curves and **(B)** fluorescence profiles of 56 clinical specimens (S1-56) by RT-qPCR and FCV-Cas13a-FLUOR detection respectively. NC, negative control (RNase-Free distilled water).
