# Supplementary figures and images for: CRISPR-Cas13a Based Visual Detection Assays for Feline Calicivirus Circulating in Southwest China
Source: Front Vet Sci. 2022 Jul 11;9:913780. doi: 10.3389/fvets.2022.913780 (PMC9310557; doi:10.3389/fvets.2022.913780)

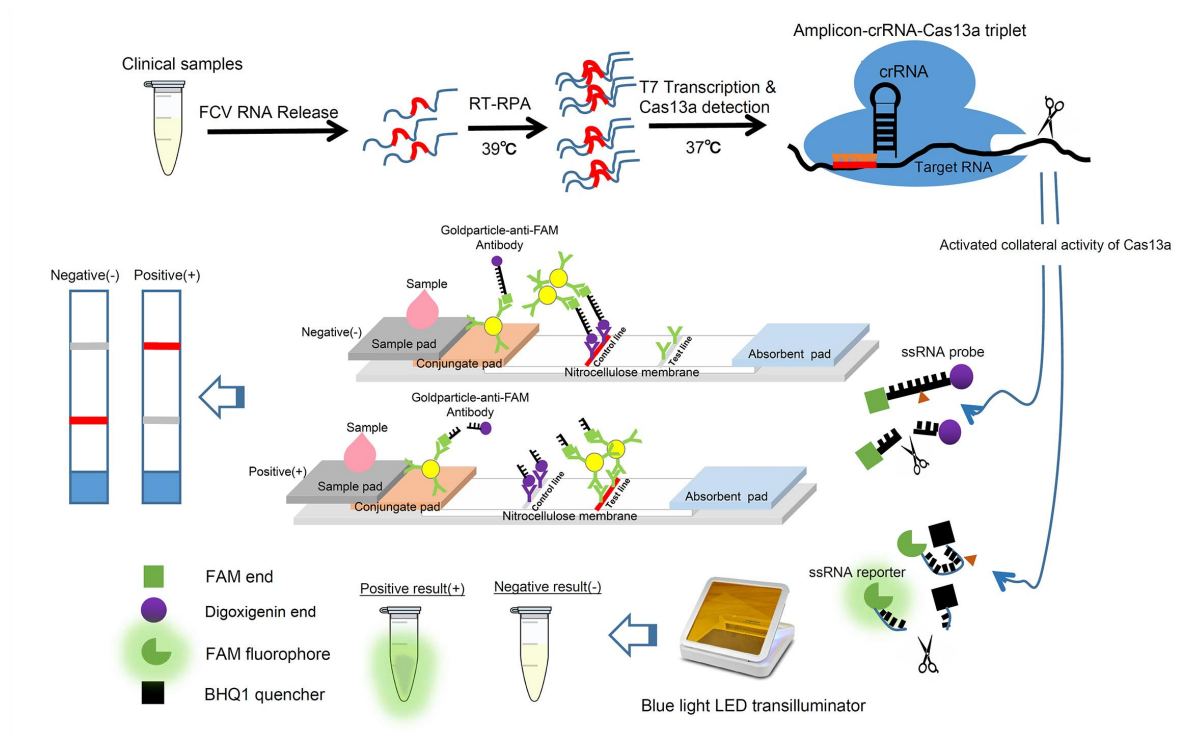

**Supplementary Figure 1.** Schematic chart of FCV-Cas13a assays (FCV-Cas13a-LFD and FCV-Cas13a-FLUOR).

Supplement: Supplementary file 3 [file Image_1.pdf]

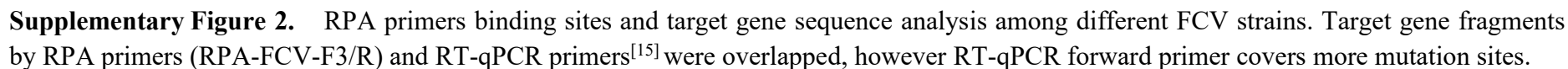

Supplement: Supplementary file 4 [file Image_2.pdf]
