# Supplementary material for: CRISPR-Cas13a Based Visual Detection Assays for Feline Calicivirus Circulating in Southwest China
Source: Front Vet Sci. 2022 Jul 11;9:913780. doi: 10.3389/fvets.2022.913780 (PMC9310557; doi:10.3389/fvets.2022.913780)
Supplement: Supplementary file 5 [file Image_3.pdf]

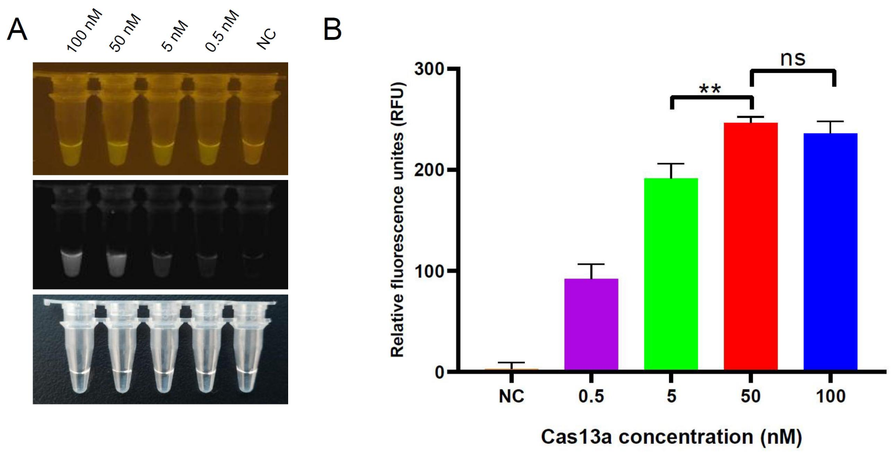

Supplementary Figure 3. (A) Screen optimal concentration of Cas13a in RSs with low concentration of plasmid DNA (55 copies/μl) using FCV-Cas13a-FLUOR. . **(B)** Endpoint fluorescence intensity of RSs with tested concentrations of Cas13a. Each experiment was repeated three times. NC, negative control; ** $p < 0.01$; ns, not significant.
