# Supplementary material for: CRISPR-Cas13a Based Visual Detection Assays for Feline Calicivirus Circulating in Southwest China
Source: Front Vet Sci. 2022 Jul 11;9:913780. doi: 10.3389/fvets.2022.913780 (PMC9310557; doi:10.3389/fvets.2022.913780)
Supplement: Supplementary file 7 [file Image_5.pdf]

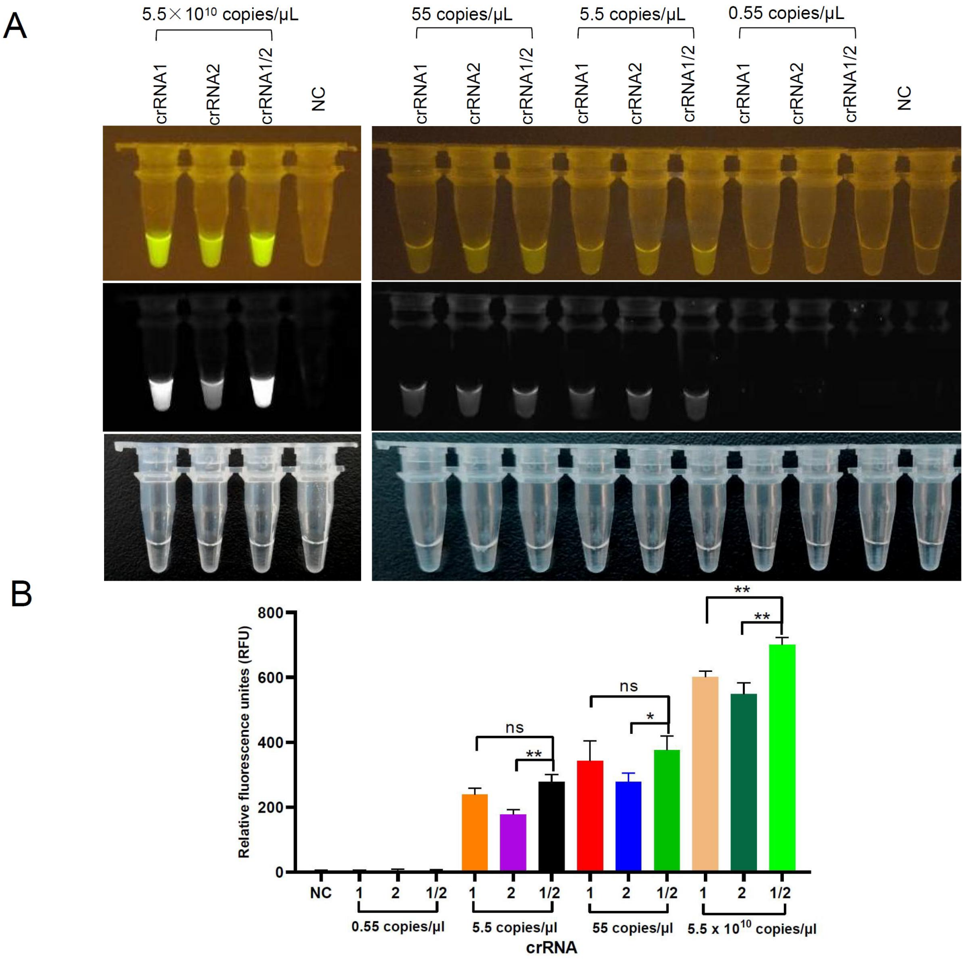

Supplementary Figure 5. (A) The testing of crRNAs efficiency using FCV-Cas13a-FLUOR in RSs with decreaing plasmid DNA concentration. The activity of dual crRNAs (crRNA1/2) was more robust than that of crRNA1 and crRNA2 respectively, but was in plasmid DNA concentration-dependent manner. **(B)** Endpoint fluorescence intensity of RSs with tested crRNAs. Each experiment was repeated three times. NC, negative control; * $p < 0.05$; ** $p < 0.01$; ns, not significant.
