# Supplementary material for: CRISPR-Cas13a Based Visual Detection Assays for Feline Calicivirus Circulating in Southwest China
Source: Front Vet Sci. 2022 Jul 11;9:913780. doi: 10.3389/fvets.2022.913780 (PMC9310557; doi:10.3389/fvets.2022.913780)
Supplement: Supplementary file 9 [file Image_7.pdf]

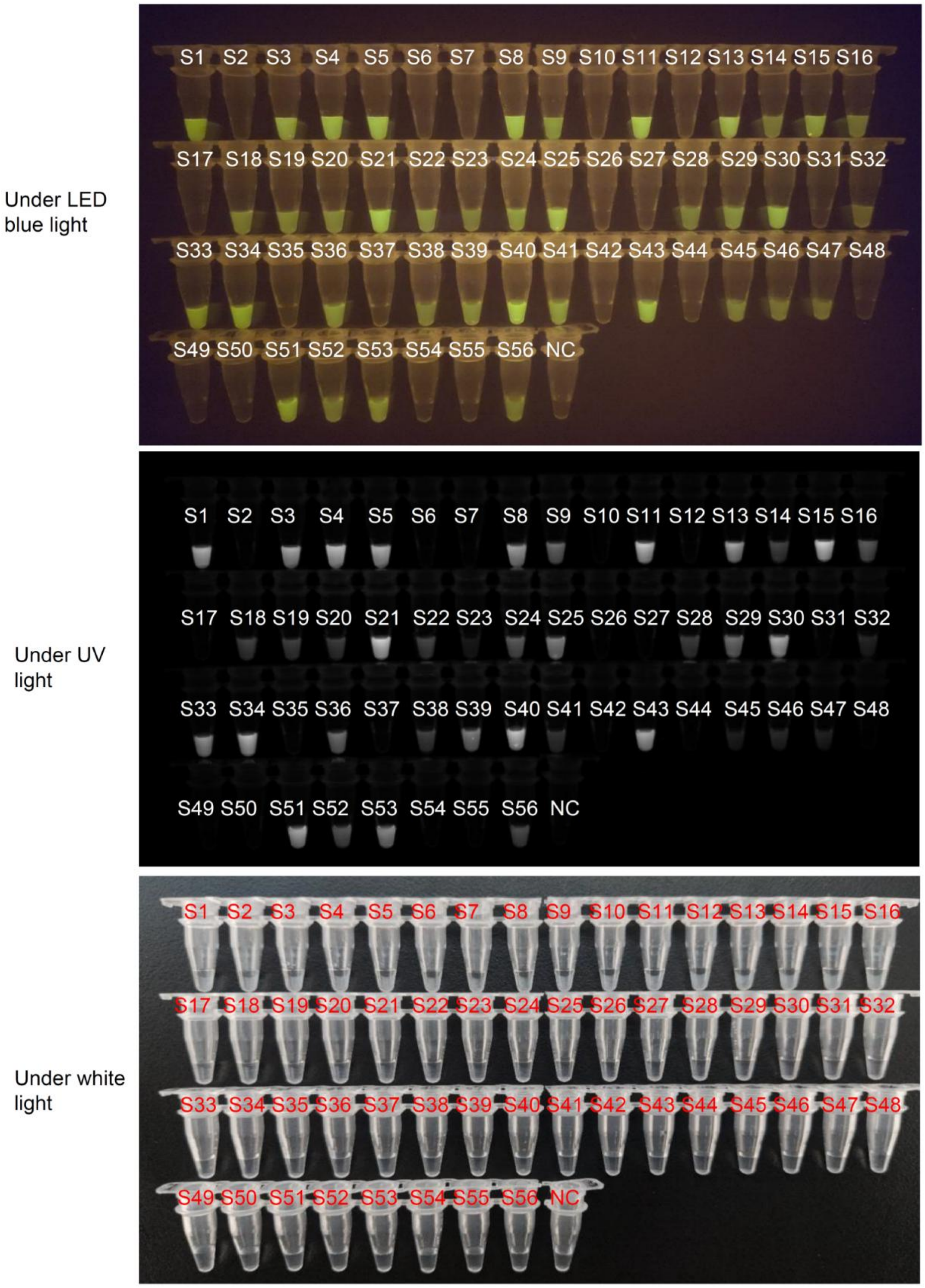

Supplementary Figure 7. Visual detection of 56 clinical samples (S1-56) usin g FCV-Cas13a-FLUOR. NC, negative control.
